# Tissue Factor Structure and Function

**DOI:** 10.6064/2012/964862

**Published:** 2012-12-26

**Authors:** Saulius Butenas

**Affiliations:** Department of Biochemistry, University of Vermont, 208 South Park Drive, Room 235A, Colchester, VT 05446, USA

## Abstract

Tissue factor (TF) is an integral membrane protein that is essential to life. It is a component of the factor VIIa-TF complex enzyme and plays a primary role in both normal hemostasis and thrombosis. With a vascular injury, TF becomes exposed to blood and binds plasma factor VIIa, and the resulting complex initiates a series of enzymatic reactions leading to clot formation and vascular sealing. Many cells, both healthy, and tumor cells, produce detectable amounts of TF, especially when they are stimulated by various agents. Despite the relative simplicity and small size of TF, there are numerous contradictory reports about the synthesis and presentation of TF on blood cells and circulation in normal blood either on microparticles or as a soluble protein. Another subject of controversy is related to the structure/function of TF. It has been almost commonly accepted that cell-surface-associated TF has low (if any) activity, that is, is “encrypted” and requires specific conditions/reagents to become active, that is, “decrypted.” However there is a lack of agreement related to the mechanism and processes leading to alterations in TF function. In this paper TF structure, presentation, and function, and controversies concerning these features are discussed.

## 1. Introduction

**Figure 1:**
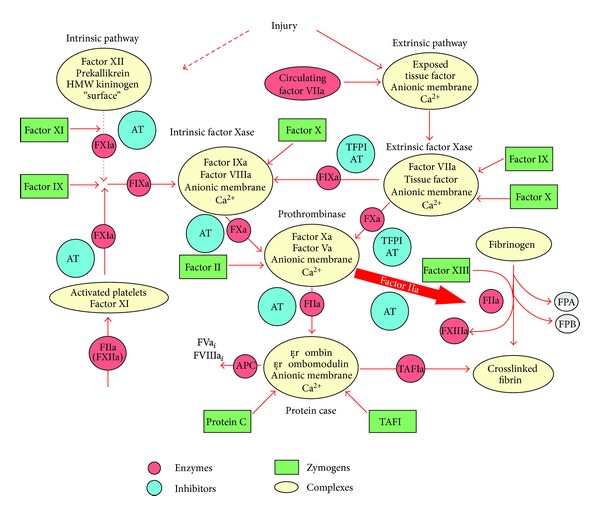
Overview of hemostasis. There are two pathways to initiate coagulation: the primary extrinsic pathway (shown on the right) and the intrinsic (also called the contact pathway) (shown on the left). The components of these multistep processes are illustrated as follows: enzymes (pink circles), inhibitors (blue circles), zymogens (green boxes), or complexes (cream ovals). The intrinsic pathway has no known bleeding etiology associated with it; thus, this pathway is considered accessory to hemostasis. Upon an injury to the vessel wall, TF, a membrane-bound cofactor, is exposed to circulating factor VIIa, forming the extrinsic factor Xase, a complex enzyme. Factor IX and factor X are converted to the serine proteases factor IXa and factor Xa, which are the enzymatic components of the intrinsic factor Xase and the prothrombinase complexes, respectively. The combined action of all three complexes lead to an explosive burst of thrombin (FIIa). Once thrombin is generated, it cleaves fibrinogen (releasing fibrinopeptides A and B (FPA and FPB, resp.)) and activates factor XIII to form a cross-linked fibrin clot. Thrombin-thrombomodulin also activates thrombin-activatable fibrinolysis inhibitor (TAFI) that slows down fibrin degradation by plasmin. Thrombin has also been shown to activate factor XI. In addition to its multiple procoagulant roles, thrombin also acts as an anticoagulant when combined with the cofactor thrombomodulin in the protein Case complex. The product of the protein Case reaction, activated protein C (APC), inactivates the cofactors factors Va and VIIIa. The cleaved species, factors Va_*i*_ (FVa_*i*_) and VIIIa_i_ (FVIIIa_*i*_), no longer support the respective procoagulant activities of the prothrombinase and the intrinsic factor Xase complexes. TF-triggered procoagulant response is also down-regulated by the stoichiometric inhibitors tissue factor pathway inhibitor (TFPI) and antithrombin (AT). TFPI serves to attenuate the activity of the extrinsic factor Xase, the trigger of coagulation. AT directly inhibits thrombin, factor IXa, and factor Xa. High molecular weight (HMW) kininogen is one of components of the intrinsic pathway. (An original version of this figure was published in Wintrobe's Clinical Hematology) [8].

TF is an integral transmembrane protein expressed by various cells, is a component of the factor VIIa-TF complex enzyme and is essential for normal hemostasis [1, 2]. Under normal circumstances cells in contact with blood do not express physiologically active TF [3]. When mechanical or chemical damage of the vascular wall occurs, subendothelial TF is expressed/exposed to blood flow and binds plasma factor VIIa, which circulates as an enzyme at a concentration of approximately 0.1 nM (1% of plasma factor VII) [4] and escapes the inhibition by serine proteases inhibitors because of its poor enzymatic qualities [1, 5]. The factor VIIa-TF complex initiates blood coagulation by activating the zymogens factor IX and factor X to their respective serine proteases, factor IXa and factor Xa. Factor IXa and factor Xa form complex enzymes with their nonenzymatic co-factors (factor VIIIa and factor Va, resp.) on the surface of membranes containing acidic phospholipids, robustly producing thrombin, the final enzymatic product of the process. Thrombin accelerates its own generation via several feed-back reactions, cleaves fibrinogen, and activates factor XIII, which leads to the formation of a crosslinked insoluble fibrin clot [6, 7] (Figure 1).

Current literature supports the notion that blood coagulation reactions are driven by the enzymatic complexes consisting of the vitamin K-dependent serine protease and a non-enzymatic cofactor assembled on a membrane surface in a calcium-dependent manner [13, 14]. The significance of such complexes could be illustrated by the alterations in catalytic efficiency displayed in proteolysis of their natural substrates. For example, the proteolytic efficiency of factor VIIa in the absence of TF is negligible [15, 16]. The addition of the cofactor, TF, leads to the assembly of a potent enzymatic complex, which proteolyses factor X at an approximately 2 · 10^7^-fold higher rate than factor VIIa alone. Similar differences in the efficiencies of enzymatic complexes versus those of enzymes alone were observed for the factor IXa-factor VIIIa complex and the factor Xa-factor Va complex [15, 17]. The significance of the membrane surface (cell or artificial) in the assembly of these complexes should not be overlooked, since in the absence of membrane the formation of the complex is either abolished or impaired [17–20]. Similarly, calcium is also an essential component for efficient complex formation [21].

The observation that tissue extracts, especially brain extracts, play an important role in activation of blood coagulation was reported already in the middle of the nineteenth century [22]. In the late eighteen hundreds, it was identified that the substance responsible for this effect is a phospholipin-protein complex [23]. The role for TF as the blood coagulation trigger (then named thromboplastin) was assigned by Loeb and Morawitz in the early twentieth century and it was found to be expressed in most animal tissues and to be released upon tissue injury [24, 25]. Over the next decades, the new findings in the field of hemostasis led to the elucidation of the coagulation cascade and its key players. The coagulation trigger, now commonly known as TF, was isolated by Nemerson's group in 1981 [26], which enabled the cloning of the protein and gene sequencing [27] and expression of recombinant forms of the protein. Since then, important roles of TF in hemostasis, thrombosis, cancer, inflammation, angiogenesis and embryogenesis have been established [28]. However, in spite of dramatically expanding knowledge related to TF, multiple controversies regarding the structure/function relationship of this protein remain subjects of scientific publications.

## 2. Structure

**Figure 2:**
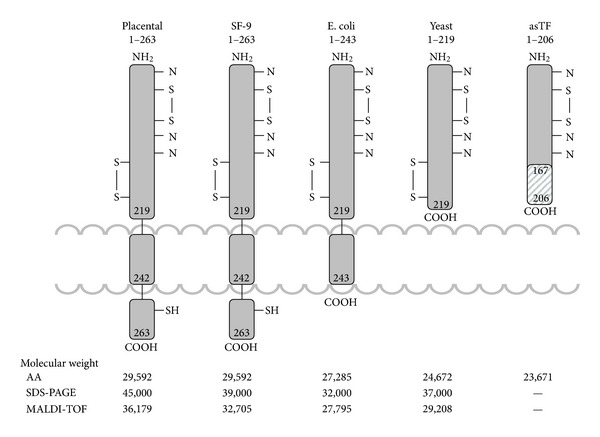
The structure of various TF species. Indicated molecular weights were determined from the amino acid composition (AA), gel electrophoresis (SDS), and mass-spectrometry (MALDI-TOF). (This figure was originally published in Surgery) [9].

TF is a 263/261 amino acid transmembrane protein containing three domains (Figure 2): (1) an extracellular domain (residues 1–219) representing the NH_2_-terminal part of the molecule composed of two fibronectin type III domains. It is involved in complex formation with factor VIIa and increases, in a membrane dependent fashion, the activity of the protease toward its natural substrates factor IX, factor X, and factor VII by several orders of magnitude [29, 30]; (2) a transmembrane domain (residues 220–242), which anchors TF to the membrane; and (3) a cytoplasmic COOH-terminal domain (residues 243–263) [27], which is involved in signal transduction [31–33].

TF binds factor VIIa with relatively high affinity, although reported dissociation constants for the factor VIIa-TF interaction vary over a wide range (from 1 pM to 20 nM) [34, 35]. TF binding to factor VIIa increases the amidolytic activity of this enzyme by approximately two orders of magnitude for small molecular weight synthetic substrates [36]. This activity is primarily dependent upon the structure of the substrate and is not influenced by the binding of TF to the membrane [1]. In contrast, to express maximum proteolytic activity toward natural substrates factor IX, factor X, and factor VII, the factor VIIa-TF complex must be formed on the surface of an appropriate membrane [29, 30]. Thus, two of the three domains of TF (extracellular and transmembrane) play distinct roles in the blood coagulation process. It has been generally accepted that TF lacking the cytoplasmic domain is functionally identical to the full-length protein in the initiation of thrombin generation. On the other hand, TF proteins lacking both the cytoplasmic and transmembrane domains cannot bind to the membrane, and therefore, while forming a complex with factor VIIa, are not efficient (if active at all) in proteolyzing natural substrates factors VII, IX, and X [29, 30].

**Figure 3:**
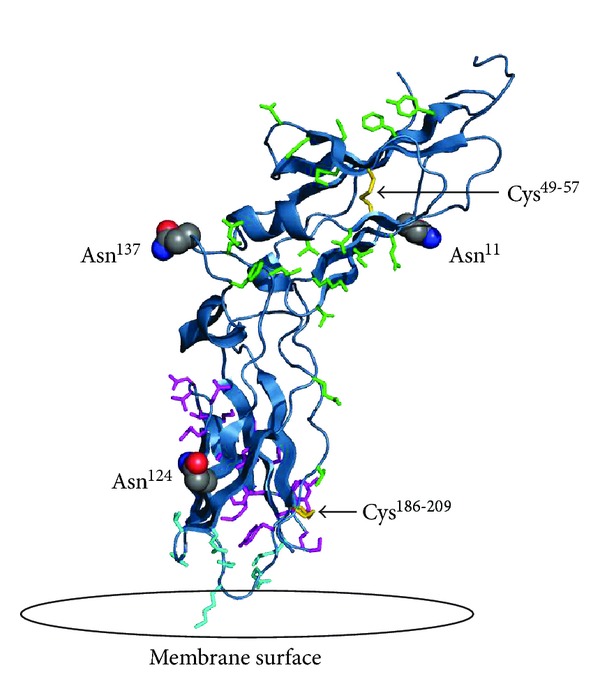
The extracellular domain of TF on a modeled lipid membrane. The figure shows in red the three sites of glycosylation (Asn^11^, Asn^124^ and Asn^137^). Highlighted in green are residues important for TF interaction with factor VIIa (Thr^17^, Lys^20^, Ile^22^, Glu^24^, Gln^37^, Asp^44^, Lys^46^, Lys^48^, Asp^58^, Thr^60^, Phe^76^, Tyr^78^, Gln^110^, Leu^133^, Arg^135^, Phe^140^, and Val^207^). Highlighted in magenta are residues important for the interaction with factor X (Thr^154^-Glu^174^ and Tyr^185^). Highlighted in aqua are the residues important for TF interaction with the membrane (Gln^118^, Val^119^, Thr^121^, Lys^159^, Asp^180^, Lys^181^, and Glu^183^). Also shown in yellow are the two disulfide bridges of TF at positions Cys^49^-Cys^57^ and Cys^186^- Cys^209^. (This figure was originally published in Biochim Biophys Acta) [10].

The gene of human TF is located on chromosome 1 p21-22 spanning approximately 12.4 kilobases. The amino acid sequence of TF has been determined from the cloned 2.3 kilobase cDNA, containing 263 amino acid residues after the cleavage of a 32 amino acid residue leader sequence [27, 37–40]. The coding sequence is made up of six exons. Exon one corresponds to the propeptide and the translation initiation sites. Exons two to five contain sites for the translation of the extracellular domain of the molecule and exon six constitutes transmembrane and cytoplasmic domains [41]. The analysis of the TF sequence revealed a distant homology to the cytokine receptor superfamily [42]. The primary sequence, as well as structural homology, places TF with a group of receptors such as the human growth hormone receptor, prolactin, erythropoietin receptor, the interferon-*γ* receptor, CD2, and CD4 [43–48]. The comparison of the three dimensional structure of the extracellular regions of these proteins and that of TF shows a domain formed by two immunoglobulin like modules. The amino (extracellular) terminus of TF (residues 1–219) is composed of two domains joined at an angle of 125 degrees [48]. The cleft formed at the interface between the two immunoglobulin-like modules was predicted to serve as the ligand binding site (Figure 3).

TF is a member of the class two cytokine receptor superfamily and fibronectin type III family. The cytokine receptor superfamily comprises a diverse group of proteins with highly homologous binding domains. The binding domains contain an approximately 200 amino acid segment with conserved regions of beta-strands. The structural analysis shows a homology in sequence and structure topology with characteristic antiparallel beta-sandwich fold with a Greek key motif. These motifs are found in the extracellular domains of a subgroup of receptor family proteins such as interferon-*α*/*β* and *γ* receptors and TF. The motif is also linked to the immunoglobulin superfamily with the analogous antiparallel *β*-sandwich topology [42, 49].

**Figure 4:**
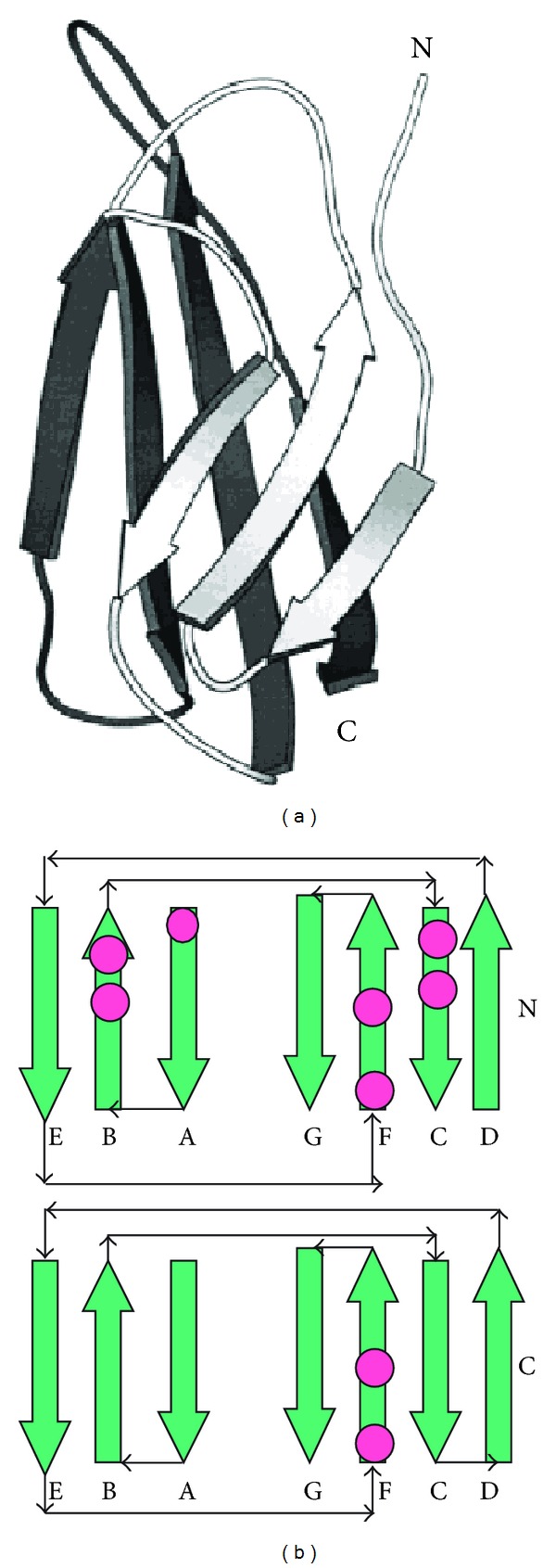
(a) Schematic presentation of fibronectin III type toplogy consisting of seven antiparallel beta-sheets. (b) Presentation of fibronectin III type N-and C-domains showing connectivity of the antiparallel beta sheets and their arrangement into a sandwich Greek key motif. The N- and C-domains constitute the extracellular domain of fibronectin III type proteins including TF.

Fibronectins are glycoproteins involved in numerous cellular processes such as blood coagulation, tissue repair, cell differentiation and embryogenesis, and so forth. The wide spectrum of activities of these molecules in signaling and binding explains their interaction with various ligands including collagen, DNA, heparin, actins, fibrin, and cytokine receptors on cell surfaces [42, 50]. Fibronectin is composed of three types of homologous repeating modules, with the type III module being the most abundant. This region in fibronectins is composed of approximately 100 amino acid residues. The extracellular domain of TF contains two type III modules. Each module is composed of two overlapping beta sheets with the top sheet containing three antiparallel beta strands and the bottom sheet containing four beta strands [42, 49–52]. The strands are connected by *β*-loops between strand *β*A and *β*B, *β*C and *β*D, *β*E and *β*F, all of which are conserved in conformations in the two modules. There are three short alpha helix segments connecting the *β*-strands. A unique feature to TF is a 17 residue *β* hairpin between strand *β*10 and strand *β*11, which is not a common element of the fibronectin superfamily. The N-terminal domain also contains a loop, an insertion of 12 residues between *β*_6_F and *β*_7_G, which is not seen in the C-terminal domain and is unique to TF [48] (Figure 4).

 Sequences of proteins with fibronectin III modules are diverse, however superposition of structures shows a conserved backbone conformation and core packing [52]. Nevertheless, some residues are specific for TF. They include a conserved Trp14 residue of strand *β*_1_C which points toward the hydrophobic core in the interface of the two domains, not conserved within the cytokine receptor superfamily. Its side chain is accommodated by Ser97 of strand *β*_7_G Pro98 found between *β*_7_G and *β*_8_A of the C-terminal domain. Asn137 of the *β* turn between strand *β*_11_ and *β*_12_C is found in human TF but is substituted by a glycine in rabbit, rat, and mouse protein, suggesting a specific role for this residue in humans.

Based on the distant sequence similarities, topology and a receptor like function, TF is also characterized as a member of the cytokine/hematopoietic growth factor receptor family. Proteins of this family are cell-surface molecules with a single transmembrane domain and a cytoplasmic domain with structural diversity. The unique characteristic of this family is a distinct disulfide bond in the extracellular domain further dividing the group into class 1 receptors and class 2 receptors with the latter including tissue factor [53].

## 3. Presentation

TF is constitutively expressed by cells associated with the vessel wall including vascular smooth muscle cells, adventitial fibroblasts and pericytes [54, 55]. At physiologic conditions, relatively high levels of TF are found specifically in the astrocytes of brain tissue, epithelial cells of the lung and cardiomyocytes of the heart and endothelial cells of placenta [56–59]. Many healthy cells produce detectable amounts of TF when they are stimulated by various agents [57–64]. TF has also been known to be expressed by tumor and tumor-like cells, where it is related to the metastatic potential of those cells [65–69]. Furthermore, TF has been identified in atherosclerotic plaques, which has suggested a role for TF in the progression of cardiovascular disease [70, 71]. However the concentration of TF in tissues and cells is low, which makes it difficult to detect, quantify and purify enough natural TF for the characterization and use in research and clinical laboratories.

**Figure 5:**
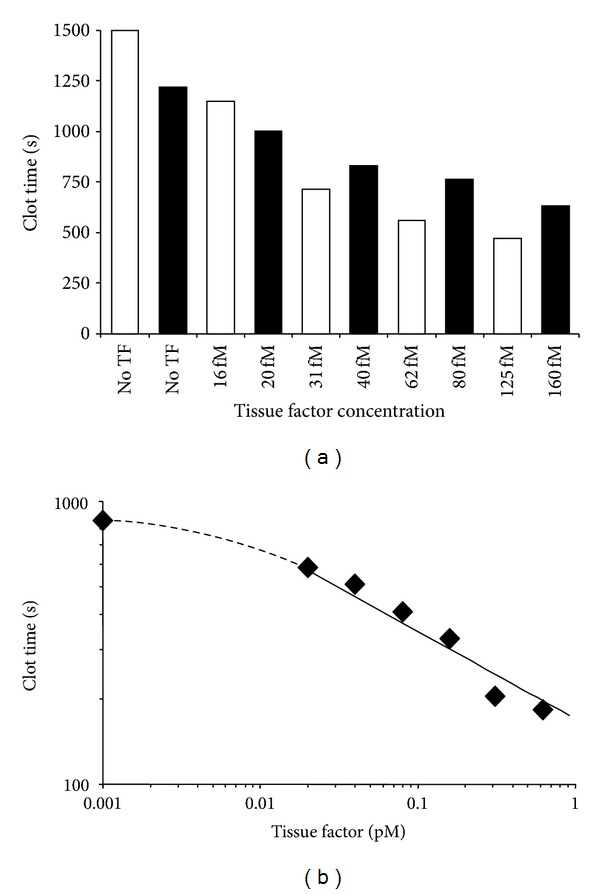
TF titrations in contact pathway inhibited whole blood (a) and plasma (b) from healthy individuals. Black and white bars represent two healthy donors. (This figure was originally published in Blood) [11].

During the last ten years, numerous conflicting studies related to the presence, concentration, and functional activity of TF circulating in blood as a soluble protein and on/in various blood cells and platelets have been published. Several groups of investigators reported the presence of TF antigen circulating in blood at the concentrations as high as 5–10 nM [72] and those of active protein reaching (sub)nanomolar concentrations [73]. In contrast, data published by several other groups indicate that if there is TF-related activity either in blood or plasma from healthy humans, the concentration of active protein does not exceed 20 fM [11, 74, 75]. Moreover, based upon the experience accumulated in several laboratories, blood or plasma activated with (sub)picomolar concentrations of functional TF clots within several minutes [76–80] (Figure 5).

It has been reported that the blood-borne TF is located on blood cells, platelets, and microparticles or that it circulates as a soluble protein. There is a common agreement that stimulation of circulating monocytes with lipopolysaccharides induces TF expression *in vitro* and *in vivo* [81–83]. It has also been shown that the expression of monocyte and monocyte-derived macrophage TF could be induced by oxidized low density lipoprotein [84, 85]. As a consequence, an increased TF-related procoagulant activity was observed. Similarly, the stimulation of monocytes through inflammatory pathways also leads to TF expression at low picomolar concentrations [86]. This expression of TF by monocytes could be enhanced by the presence of platelet-monocyte aggregates [87].

The existence of TF on platelets has been controversial and is still unresolved. The sources of hypothetical TF in platelets have been described to include denovo synthesis and storage in *α*-granules as well as absorption of monocyte-shed TF-containing microparticles [88, 89]. Studies by Zillmann et al. suggested that platelets isolated from stimulated blood contain functional TF [90] and Müller et al. claimed the presence of TF in *α*-granules of resting platelets [91]. Panes and coworkers believed that platelets synthesize TF in response to activation [92] and other studies suggested the presence of TF mRNA in platelets [93–96]. These suggestions have been challenged by our data based on the observation that no detectable TF activity or antigen are detected either on resting and ionophore-treated washed platelets or lipopolysaccharide-treated blood platelets [11, 97]. Similarly, Osterud and coworkers failed to detect any TF activity on collagen stimulated platelets [98, 99]. Bouchard and coworkers also did not observe TF expression by human platelets stimulated with PAR-1 and PAR-4 agonist peptides [97]. In contrast to Camera et al. transient TF expression was not detected upon platelet stimulation for a short (15 min) time [100, 101].

Similar to the subject of platelet TF, there is little agreement related to the presence of TF on granulocytes. Maugeri et al. suggested that granulocytes produce TF upon stimulation [102] while other authors have reported the expression of TF in neutrophils [103] and eosinophils [104]. However, data from Osterud's laboratory show no evidence of TF expression in any granulocytic cells [105–107].

Microparticles, small anucleoid cell membrane fragments, are released by various cells upon their stimulation or during cell apoptosis or death [108]. Microparticles are quite heterogenous with respect to size (usually from 100 to 1000 nm) and membrane lipid and protein composition, all of them dependent upon the microparticles' cellular origin and their generation pathway [109]. It is predictable that microparticles shed by the TF-bearing cells would contain TF on their surface. However, due to the controversy related to the presence of TF on some cell types and platelets, the presence of TF on microparticles shed by those cells remains a subject of the discussion. For example, while the role of lipopolysaccharide-stimulated monocytes as a source of microparticles containing active TF has been established [98, 110–112], that of platelets and granulocytes remains questionable [110, 113–115]. Despite the controversies related to sources of TF-bearing microparticles, there has been a growing body of evidence that this form of TF is associated with pathologic conditions, such as pulmonary embolism [116], venous thromboembolism [117], and disseminated intravascular coagulation [118], particularly in patients with various types of malignancy [118–122]. On the bright side, microparticle TF has a potential to promote hemostasis in hemophilia situation [123].

The presence, source, and function of a soluble form of alternatively spliced TF in blood have also been subjects of controversy [124–127]. It has been suggested that this form of TF is procoagulant [128] and stimulates clot growth [124]. However subsequent studies showed that alternatively spliced TF has no procoagulant activity [125–127] but that it could protect cells from apoptosis [129] and promote tumor growth and angiogenesis [126, 130–132]. Alternatively, Khan et al. suggested that soluble TF can bind to peripheral monocytes and platelets and efficiently activate factor VII [133]. The potential origin of this discrepancy could be assigned to the physiologically irrelevant conditions [3, 124] used and the lack of validated commercial assays for the detection of alternatively spliced TF activity at its physiologic concentrations [134–136]. TF antigen in blood may also be detected as a degradation product and not necessarily as an alternatively spliced form.

The majority of studies reporting high concentrations of TF in plasma and the presence of TF in platelets and blood cells use commercial assays. For example, in a study by Bis et al. which used a commercial TF assay, nanomolar concentrations of TF in plasma from patients with acute coronary syndrome a were reported [137]. Using validated assays for the quantitation of TF antigen [135] and activity [138] developed in our laboratory, we found that the TF antigen concentrations in plasmas from patients with a similar diagnosis are at low picomolar levels, with an average functional concentration less than 0.4 pM [138]. Until there is agreement in the scientific community concerning the validity of the assays used by various laboratories, incongruent reports will continue to accumulate in the literature.

## 4. Posttranslational Modifications

While contributions of various regions of the primary structure of TF on its activity are relatively well established, the data related to the influence of posttranslational modifications on the function of TF are scarce, if available at all, primarily due to the shortage of natural TF protein. To compensate for this shortage, a variety of human recombinant TF species have been produced in different expression systems [27, 30, 139]. These recombinant proteins have been extensively used worldwide, and the experimental results acquired using recombinant proteins in vitroare frequently extrapolated to coagulation processes occurring in vivo. It has been commonly accepted that recombinant TF proteins are functionally identical to natural TF [140]. Unfortunately, the nonavailability of isolated natural TF does not allow the confirmation (or rejection) of results obtained with recombinant proteins or with those present in homogenates of natural tissues. Additionally, it leads to a scarcity of data addressing the influence of some structural components of natural TF on its activity. As a consequence, there is plenty of controversy in published studies related to the structure/activity of natural TF. A major obstacle arises as to the protein's genuine folding, activity, and function caused by different posttranslational modifications as per specific expression system. Limited work has been done on the contribution of each modification to the activity of TF, although recently more attention has been directed to the subject of structure-function relationship of natural TF [12].

According to the mass-spectrometry data, the level of posttranslational modifications of various forms of TF varies from 377 Da for recombinant TF 1–243 to 6,605 Da for the natural placental TF protein [12, 136]. Recombinant TF 1–263 is less modified (3,604 Da) than placental (Figure 2).

It was predictable that the prevailing posttranslational modification of TF would be related to carbohydrates. The amino acid sequence data indicate that full-length TF has three potential glycosylation sites at Asn^11^, Asn^124^, and Asn^137^ in the extracellular domain of the protein and one (Asn^261^) in the cytoplasmic domain [27, 141]. The Asn^261^ site is not present in the truncated TF 1–243. Already in 1944, Chargaff et al. observed the presence of carbohydrtaes in TF [142], with the suggested sugar content constituting between 7–13% of total protein mass. A thorough analysis of the carbohydrate content of TF protein by Bjorklid [143] showed a carbohydrate content of 6%, mostly composed of fucose, mannose, galactose and N-acetylneuranimidase. The linkage of carbohydrates to the TF backbone was presumed to be via asparagine. Paborsky and Harris determined three potential sites for TF glycosylation in the extracellular domain, all of which were within the recognized sequence for N-linked glycosylation, that is, N-X-T/S [141]. Based on a similar activity of carbohydrate-free recombinant TF from *E. coli* and glycosylated protein expressed by kidney cells, it was concluded that carbohydrates play no role in TF function. Rickles, Waxmann, Stone, and their coworkers [140, 144, 145] also suggested that glycosylation is not required for the function of TF. In contrast, Pitlick, Shands, Bona, and data from our laboratory demonstrated that carbohydrates play a considerable role in TF activity [12, 146–148]. Pitlick observed that concanavalin A inhibits the coagulant activity of TF by binding reversibly to the carbohydrate moiety of the protein [146]. Shands and Bona both observed the loss of function and inability of TF to be incorporated into membranes after treatment with tunicamycin [147, 148].

**Figure 6:**
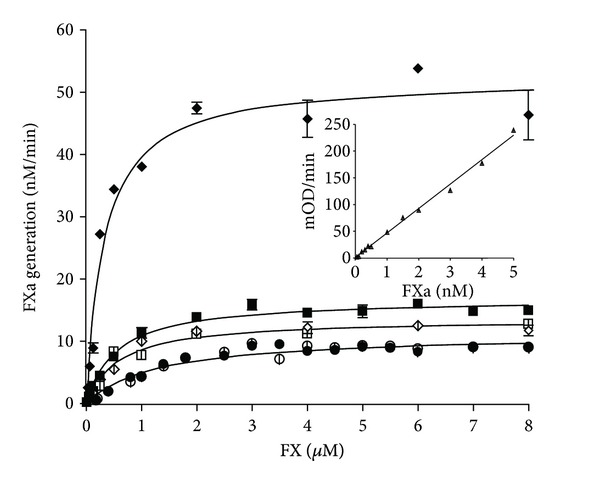
Proteolytic activity of the extrinsic factor Xase formed by factor VIIa and relipidated placental TF (*◆* glycosylated and *⋄* deglycosylated), full-length recombinant TF 1–263 (■ glycosylated and □ deglycosylated) and truncated recombinant TF 1–243 (*∙* native and ° “deglycosylated”). Factor VIIa (5 nM) was incubated with 0.1 nM relipidated TF and increasing concentrations of factor X were added. Factor Xa generation was monitored in a chromogenic assay. (This figure was originally published in J Biol Chem) [12].

A direct evidence for the effect of glycosylation on TF function came from our laboratory when we compared carbohydrate-free recombinant TF 1–243 expressed in *E.coli,* glycosylated recombinant TF 1–263 from Sf9 insect cells and natural TF from human placenta. Deglycosylated forms of the latter two proteins were included into analysis as well. The extent of glycosylation and structure of carbohydrates at each potential glycosylation site of all three TF proteins were somewhat different. No carbohydrates were detected on recombinant TF 1–243 produced in *E. coli *[12]. All three TF proteins analyzed were tested for their effect on FVIIa activity. For recombinant TF 1–263, deglycosylation had little effect on the affinity for FVIIa and it only marginally decreased the activity of the formed factor VIIa-TF complex. A more pronounced effect of deglycosylation was observed for placental TF. Deglycosylation significantly decreased the catalytic efficiency of the factor VIIa-TF complex towards the natural substrate factor X [12]. After deglycosylation, the catalytic efficiency of factor Xa generation became comparable for the placental, recombinant 1–263 and native recombinant TF 1–243 (Figure 6). Analysis of the relative carbohydrate abundance revealed that out of the four potential sites for N-linked glycosylation, two (Asn^124^ and Asn^137^) were found to undergo complete glycosylation in both recombinant 1–263 and placental TF proteins [10]. An incomplete glycosylation occurs at Asn^11^ of recombinant TF 1–263 with a relative abundance of carbohydrates of 20%, whereas the glycosylation on Asn^11^ of placental TF reaches 76%. No carbohydrates were found at Asn^261^ in either protein. The composition of carbohydrates varied between these two proteins and between each site within each protein. At all three glycosylation sites, recombinant TF 1–263 predominantly contains high mannose sugars, whereas natural placental protein contains either hybrid or complex carbohydrates with high mannose sugars absent. A unique characteristic of placental TF is the presence of sialic acid on all three glycosylation sites. Thus in contrast to previously published statements that posttranslational modifications have no effect on TF activity [140, 141], these data indicate that glycosylation and the structure of carbohydrates have a pronounced effect on TF function.

Phosphorylation is another important posttranslational modification because it plays a critical role in the regulation of many protein functions. Upon protein phosphorylation, the phosphate is transferred to the hydroxyl groups of the side chains of three amino acids—serine, threonine, and tyrosine [149], with the hydroxyl groups of serine representing the major site of protein phosphorylation (90–95% of total phosphorylation sites). In 1992, Zioncheck et al. determined that TF contains two phosphorylation sites, both of them located in the cytoplasmic domain [150]. From the alignment of cDNA sequences of several TF species (including human) it was concluded that phosphorylation sites contain a conserved amino acid sequence X-Ser*/Thr*-Pro-X with the asterisk indicating the phosphorylation residue. In a later publication, Mody and Carson suggested that the cytoplasmic domain of TF can be phosphorylated in vitro at multiple sites, particularly at Ser^253^ and Ser^258^ [151]. The mutational data presented by Dorfleutner and Ruf suggested that initial phosphorylation at Ser^253^ enhances the subsequent phosphorylation at Ser^258^ [152]. In several publications the influence of phosphorylation on cell TF activity has been suggested [153], primarily by altering TF expression [154] and cell signaling, migration and angiogenesis [31, 155, 156]. Rydén and coworkers showed that TF phosphorylation related protease-activated receptor-2 signaling plays an important role in breast cancer recurrence [157].

Another common posttranslational modification of eukaryotic proteins is acylation with a fatty acid palmitate, which occurrs at a cysteine residue via a thioester bond formation [158]. S-palmitoylation is almost an exclusive feature of membrane proteins, although there is no well-defined sequence for this modification other than the presence of a free cysteine. S-palmitoylation occurs in the vicinity of cell membranes and directs proteins to the membrane lipid rafts, presumably due to a high affinity of proteins modified with fatty acids for these subdomains of cell membranes [159]. It has been shown in a study by Bach and coworkers that TF has one S-palmitoylation site in the intracellular domain of the protein at Cys^245^ [160]. The Cys^245^ is located at the amino terminus of the intracellular domain and close to the membrane surface. The extent of palmitoylation at this site, however, is not clear because it has been shown in several publications that in purified TF proteins, Cys^245^ can also participate in an inter-molecular disulfide bond formation [160–163]. It has been suggested that S-palmitoylated TF should target cell membrane's lipid rafts, which are enriched in sphingolipids and cholesterol [164]. Increasing experimental data suggest a role for these rafts in modification of tissue factor expression [165, 166] and activity [167–170], although the latter subject remains controversial [171]. Another mechanism for TF activity regulation by S-palmitoylation is based upon its effect on the phosphorylation of the intracellular domain of the protein. Dorfleutner and Ruf have shown that S-palmitoylation at Cys^245^ inhibits phosphorylation at Ser^258^ in the intracellular domain of TF [152]. It has been suggested by the authors that palmitoylation favors the association of TF with caveolin-containing lipid rafts. Thus, S-palmitoylation can indirectly alter the procoagulant activity of TF by influencing phosphorylation of the intracellular domain.

## 5. Cysteines and Disulfides

A quite popular hypothesis describing cell TF activity suggests that many cells, including those in contact with blood, contain under normal physiologic conditions inactive, that is, “encrypted” TF on their surface and that it needs “decryption” to express the procoagulant activity [172]. Several, often contradictory mechanisms have been hypothesized in attempts to explain “encryption-decryption” of TF activity. One of the proposed mechanisms is related to the disulfide bond formation in the extracellular domain of TF.

There are four cysteines (Cys^49^, Cys^57^, Cys^186^, and Cys^209^) located in the extracellular domain of TF [160], which can potentially form disulfide bonds. The carboxyterminal cytoplasmic domain of TF contains a single Cys^245^ residue, which is acylated. Two disulfide bridges between Cys^49^–Cys^57^ and Cys^186^–Cys^209^ have been reported [173]. The role of the latter (Cys^186^–Cys^209^) in the regulation of TF function and the mechanism by which it is formed on the cell surface has been the subject of debates for the last years [174–189]. The suggested range of importance for this bond is from essential [180, 189] to not having any effect on TF function [186, 188]. Bach and coworkers suggested in 1981 that preservation of disulfides is necessary for TF activity [160]. Based on mutagenesis studies, a nonfunctional role has been assigned to the N-terminal disulfide Cys^49^–Cys^57^, whereas an important functional role has been assigned to the C-terminal disulfide Cys^186^–Cys^209^, because mutation of these cysteines was shown to impair the procoagulant activity of TF [173, 190]. This C-terminal cysteine bridge has been described as an allosteric disulfide bond [174]. An allosteric bond controls protein function by triggering conformational change upon its reduction or oxidation. Unlike the catalytic disulfide bond, which enzymatically mediates thiol-disulfide interchanges in substrate proteins, the allosteric bond nonenzymatically changes the protein structure [191]. Based on an observation that TF activity increased upon the treatment of cells with mercuric chloride, an oxidizing agent [174], it has been suggested that mercuric chloride can oxidize two cysteines forming a disulfide bond. Other publications, however, show that mercuric chloride oxidizes only a single thiol group [192, 193] and that a similar effect could be achieved by treating TF-bearing cells with other metal compounds [175]. Another hypothesis related to the Cys^186^–Cys^209^ disulfide bond suggests protein disulfide isomerase as a regulator of cell TF activity *via* its effect on the oxidation/reduction of this bond [176–180]. In contrast to these publications, several studies from other laboratories suggested that the TF activity-enhancing effect of protein disulfide isomerase is related to the presence of acidic phospholipids either as a contaminant in the preparations of protein disulfide isomerase [181, 182] or due to their relocation to the cell surface upon treatment with protein disulfide isomerase or mercuric chloride [183–186]. Moreover, one study suggested that Cys^186^ and Cys^209^ are not available for interaction with protein disulfide isomerase when TF at physiologically relevant conditions is bound to factor VIIa [187].

There are several possible explanations for the contradictions observed in publications describing the influence of protein disulfide isomerase on TF activity: (1) variability in reagents, procedures and cell lines used in different laboratories; and (2) the lack of specificity of protein disulfide isomerase for TF. Protein disulfide isomerase has an effect on cell membrane lipid composition [184, 185] and it targets multiple proteins in the cell, catalyzing thiol-disulfide exchange [194–196]. Both of those processes could alter TF activity without changing the status of thiols/disulfides. Additionally, protein disulfide isomerase can alter thrombin generation in a TF-independent manner via coagulation factor ligation to platelets [197] or by catalyzing complex formation with participation of thrombin and antithrombin [198].

Although the status of cysteines of the extracellular domain of TF located on the surface of resting cells remains an open question, recent data from several laboratories brings new knowledge related to their effect on TF function. In contrast to Kothari et al. who suggested that the Cys^186^–Cys^209^ disulfide bond is not essential for the cell TF procoagulant activity [188], van den Hengel and coworkers demonstrated that the absence of this bond completely abolishes function of human TF presented on the cell surface [189]. A similar conclusion has been drawn about the essential role of the Cys^190^–Cys^213^ disulfide bond for mouse TF function [199]. Mass spectrometry-based data from our laboratory indicate that the reduction of the Cys^186^–Cys^209^ bond completely eliminates TF co-factor function, despite that the reduced protein is still able to bind to the enzymatic component of the complex, factor VIIa.

## 6. Conclusions

TF is an *in vivo* initiator of blood coagulation and is essential for life. It has several types of posttranslational modifications, some of which are different for the natural and recombinant proteins. Although those modifications of TF were identified several decades ago, thorough characterization and an evaluation of their influence on TF function have been somewhat neglected. This neglection was related primarily to the scarce availability of the natural TF protein, and, as a consequence, most of the experiments were done using recombinant TF proteins, despite the known differences in structure and activity of these proteins compared to the natural TF. These differences are translated into differences in physiologically-relevant activities of TF. Thus, caution should be used in data interpretation when a recombinant protein is used as a surrogate for the natural protein, especially in diagnostic and biological experiments. Lately, the interest in TF structure-activity relationship has been rekindled, primarily by the controversies related to the role of disulfides of the extracellular domain and that of glycosylation. Additionally, there has been an increasing number of studies accomplished using natural human TF or that present on the cell surface instead of recombinant proteins. This increased interest leads us to believe that the gap existing in the knowledge related to the structure-activity relationship of TF will be complemented with new research data.

## References

[1] Lawson JH, Butenas S, Mann KG (1992). The evaluation of complex-dependent alterations in human factor VIIa. *Journal of Biological Chemistry*.

[2] Mackman N (2004). Role of tissue factor in hemostasis, thrombosis, and vascular development. *Arteriosclerosis, Thrombosis, and Vascular Biology*.

[3] Butenas S, Mann KG (2004). Active tissue factor in blood?. *Nature Medicine*.

[4] Morrissey JH, Macik BG, Neuenschwander PF, Comp PC (1993). Quantitation of activated factor VII levels in plasma using a tissue factor mutant selectively deficient in promoting factor VII activation. *Blood*.

[5] Lawson JH, Butenas S, Ribarik N, Mann KG (1993). Complex-dependent inhibition of factor VIIa by antithrombin III and heparin. *Journal of Biological Chemistry*.

[6] Mann KG, Orfeo T, Butenas S, Undas A, Brummel-Ziedins K (2009). Blood coagulation dynamics in haemostasis. *Hamostaseologie*.

[7] Butenas S, Mann KG (2002). Blood coagulation. *Biochemistry*.

[8] Brummel-Ziedins K, Orfeo T, Jenny NS, Everse SJ, Mann KG, Greer JP, Foerster J, Rodgers GM (2004). Blood coagulation and fibrinolysis. *Wintrobe'S Clinical Hematology*.

[9] Butenas S, Orfeo T, Brummel-Ziedins KE, Mann KG (2007). Tissue factor in thrombosis and hemorrhage. *Surgery*.

[10] Krudysz-Amblo J, Jennings ME, Matthews DE, Mann KG, Butenas S (2011). Differences in the fractional abundances of carbohydrates of natural and recombinant human tissue factor. *Biochimica et Biophysica Acta*.

[11] Butenas S, Bouchard BA, Brummel-Ziedins KE, Parhami-Seren B, Mann KG (2005). Tissue factor activity in whole blood. *Blood*.

[12] Krudysz-Amblo J, Jennings ME, Mann KG, Butenas S (2010). Carbohydrates and activity of natural and recombinant tissue factor. *Journal of Biological Chemistry*.

[13] Seegers WH (1949). Activation of purified prothrombin. *Proceedings of the Society for Experimental Biology and Medicine*.

[14] Mann KG, Nesheim ME, Church KR, Haley P, Krishnaswamy S (1990). Surface-dependent reactions of the vitamin K-dependent enzyme complexes. *Blood*.

[15] Komiyama Y, Pedersen AH, Kisiel W (1990). Proteolytic activation of human factors IX and X by recombinant human factor VIIa: effects of calcium, phospholipids, and tissue factor. *Biochemistry*.

[16] Bom VJJ, Bertina RM (1990). The contributions of Ca^2+^, phospholipids and tissue-factor apoprotein to the activation of human blood-coagulation factor X by activated factor VII. *Biochemical Journal*.

[17] Mann KG, Krishnaswamy S, Lawson JH (1992). Surface-dependent hemostasis. *Seminars in Hematology*.

[18] Nesheim ME, Taswell JB, Mann KG (1979). The contribution of bovine factor V and factor Va to the activity of prothrombinase. *Journal of Biological Chemistry*.

[19] Tracy PB, Nesheim ME, Mann KG (1981). Coordinate binding of factor Va and factor Xa to the unstimulated platelet. *Journal of Biological Chemistry*.

[20] Lamphear BJ, Fay PJ (1992). Factor IXa enhances reconstitution of factor VIIIa from isolated A2 subunit and A1/A3-C1-C2 dimer. *Journal of Biological Chemistry*.

[21] Nelsestuen GL, Kisiel W, Di Scipio RG (1978). Interaction of vitamin K dependent proteins with membranes. *Biochemistry*.

[22] Mills C (1921). Chemical nature of tissue coagulins. *Biochemistry*.

[23] Wooldridge L (1893). *On the Chemistry of the Blood and other Scientific Papers*.

[24] Camerer E, Kolstø AB, Prydz H (1996). Cell biology of tissue factor, the principal initiator of blood coagulation. *Thrombosis Research*.

[25] Howell W (1912). The nature and action of the thromboplastic (zymoplastic) substance of the tissues. *American Journal of Physiology*.

[26] Bach R, Nemerson Y, Konigsberg W (1981). Purification and characterization of bovine tissue factor. *Journal of Biological Chemistry*.

[27] Spicer EK, Horton R, Bloem L (1987). Isolation of cDNA clones coding for human tissue factor: primary structure of the protein and cDNA. *Proceedings of the National Academy of Sciences of the United States of America*.

[28] Mackman N (2009). The many faces of tissue factor. *Journal of Thrombosis and Haemostasis*.

[29] Ruf W, Rehemtulla A, Morrissey JH, Edgington TS (1991). Phospholipid-independent and -dependent interactions required for tissue factor receptor and cofactor function. *The Journal of Biological Chemistry*.

[30] Fiore MM, Neuenschwander PF, Morrissey JH (1994). The biochemical basis for the apparent defect of soluble mutant tissue factor in enhancing the proteolytic activities of factor VIIa. *Journal of Biological Chemistry*.

[31] Ahamed J, Ruf W (2004). Protease-activated receptor 2-dependent phosphorylation of the tissue factor cytoplasmic domain. *Journal of Biological Chemistry*.

[32] Siegbahn A, Johnell M, Sorensen BB, Petersen LC, Heldin CH (2005). Regulation of chemotaxis by the cytoplasmic domain of tissue factor. *Thrombosis and Haemostasis*.

[33] Collier MEW, Ettelaie C (2011). Regulation of the incorporation of tissue factor into microparticles by serine phosphorylation of the cytoplasmic domain of tissue factor. *Journal of Biological Chemistry*.

[34] Toomey JR, Smith KJ, Stafford DW (1991). Localization of the human tissue factor recognition determinant of human factor VIIa. *Journal of Biological Chemistry*.

[35] Schullek JR, Ruf W, Edgington TS (1994). Key ligand interface residues in tissue factor contribute independently to factor VIIa binding. *Journal of Biological Chemistry*.

[36] Butenas S, Ribarik N, Mann KG (1993). Synthetic substrates for human factor VIIa and factor VIIa-tissue factor. *Biochemistry*.

[37] Scarpati EM, Wen D, Broze GJ (1987). Human tissue factor: cDNA sequence and chromosome localization of the gene. *Biochemistry*.

[38] Kao FT, Hartz J, Horton R, Nemerson Y, Carson SD (1988). Regional assignment of human tissue factor gene (F3) to chromosome 1p21-p22. *Somatic Cell and Molecular Genetics*.

[39] Morrissey JH, Fakhrai H, Edgington TS (1987). Molecular cloning of the cDNA for tissue factor, the cellular receptor for the initiation of the coagulation protease cascade. *Cell*.

[40] Fisher KL, Gorman CM, Vehar GA, O’Brien DP, Lawn RM (1987). Cloning and expression of human tissue factor cDNA. *Thrombosis Research*.

[41] Mackman N, Morrissey JH, Fowler B, Edgington TS (1989). Complete sequence of the human tissue factor gene, a highly regulated cellular receptor that initiates the coagulation protease cascade. *Biochemistry*.

[42] Bazan JF (1990). Structural design and molecular evolution of a cytokine receptor superfamily. *Proceedings of the National Academy of Sciences of the United States of America*.

[43] de Vos AM, Ultsch M, Kossiakoff AA (1992). Human growth hormone and extracellular domain of its receptor: crystal structure of the complex. *Science*.

[44] Somers W, Ultsch M, De Vos AM, Kossiakoff AA (1994). The X-ray structure of growth hormone-prolactin receptor complex. *Nature*.

[45] Livnah O, Stura EA, Johnson DL (1996). Functional mimicry of a protein hormone by a peptide agonist: the EPO receptor complex at 2.8 Å. *Science*.

[46] Walter MR, Windsor WT, Nagabhushan TL (1995). Crystal structure of a complex between interferon-*γ* and its soluble high-affinity receptor. *Nature*.

[47] Bodian DL, Jones EY, Harlos K, Stuart DI, Davis SJ (1994). Crystal structure of the extracellular region of the human cell adhesion molecule CD2 at 2.5Å resolution. *Structure*.

[48] Harlos K, Martin DMA, O’Brien DP (1994). Crystal structure of the extracellular region of human tissue factor. *Nature*.

[49] Baron M, Main AL, Driscoll PC, Mardon HJ, Boyd J, Campbell LD (1992). 1H NMR assignment and secondary structure of the cell adhesion type III module of fibronectint. *Biochemistry*.

[50] Dean DC, Bowlus CL, Bourgeois S (1987). Cloning and analysis of the promoter region of the human fibronectin gene. *Proceedings of the National Academy of Sciences of the United States of America*.

[51] Main AL, Harvey TS, Baron M, Boyd J, Campbell ID (1992). The three-dimensional structure of the tenth type III module of fibronectin: an insight into RGD-mediated interactions. *Cell*.

[52] Muller YA, Ultsch MH, De Vos AM (1996). The crystal structure of the extracellular domain of human tissue factor refined to 1.7 Å resolution. *Journal of Molecular Biology*.

[53] Peppelenbosch MP, Versteeg HH (2001). Cell biology of tissue factor, an unusual member of the cytokine receptor family. *Trends in Cardiovascular Medicine*.

[54] Bouchard BA, Shatos MA, Tracy PB (1997). Human brain pericytes differentially regulate expression of procoagulant enzyme complexes comprising the extrinsic pathway of blood coagulation. *Arteriosclerosis, Thrombosis, and Vascular Biology*.

[55] Schecter AD, Spirn B, Rossikhina M (2000). Release of active tissue factor by human arterial smooth muscle cells. *Circulation Research*.

[56] Flossel C, Luther T, Muller M, Albrecht S, Kasper M (1994). Immunohistochemical detection of tissue factor (TF) on paraffin sections of routinely fixed human tissue. *Histochemistry*.

[57] Drake TA, Morissey JH, Edgington TS (1989). Selective cellular expression of tissue factor in human tissues. Implications for disorders of hemostasis and thrombosis. *American Journal of Pathology*.

[58] Fleck RA, Rao LVM, Rapaport SI, Varki N (1990). Localization of human tissue factor antigen by immunostaining with monospecific, polyclonal anti-human tissue factor antibody. *Thrombosis Research*.

[59] Eddleston M, de la Torre JC, Oldstone MBA, Loskutoff DJ, Edgington TS, Mackman N (1993). Astrocytes are the primary source of tissue factor in the murine central nervous system. A role for astrocytes in cerebral hemostasis. *Journal of Clinical Investigation*.

[60] Bloem LJ, Chen L, Konigsberg WH, Bach R (1989). Serum stimulation of quiescent human fibroblasts induces the synthesis of tissue factor mRNA followed by the appearance of tissue factor antigen and procoagulant activity. *Journal of Cellular Physiology*.

[61] Levi M, van der Poll T, Ten Cate H (2006). Tissue factor in infection and severe inflammation. *Seminars in Thrombosis and Hemostasis*.

[62] Bouchard BA, Tracy PB (2003). The participation of leukocytes in coagulant reactions. *Journal of Thrombosis and Haemostasis*.

[63] Nijziel M, Van Oerle R, Van’t Veer C, Van Pampus E, Lindhout T, Hamulyák K (2001). Tissue factor activity in human monocytes is regulated by plasma: implications for the high and low responder phenomenon. *British Journal of Haematology*.

[64] Broussas M, Cornillet-Lefèbvre P, Potron G, Nguyên P (2002). Adenosine inhibits tissue factor expression by LPS-stimulated human monocytes: involvement of the A3 adenosine receptor. *Thrombosis and Haemostasis*.

[65] Ando R, Kase S, Ohashi T (2011). Tissue factor expression in human pterygium. *Molecular Vision*.

[66] Edwards RL, Rickles FR, Cronlund M (1981). Abnormalities of blood coagulation in patients with cancer. Mononuclear cell tissue factor generation. *Journal of Laboratory and Clinical Medicine*.

[67] Ruf W, Mueller BM (2006). Thrombin generation and the pathogenesis of cancer. *Seminars in Thrombosis and Hemostasis*.

[68] López-Pedrera C, Barbarroja N, Dorado G, Siendones E, Velasco F (2006). Tissue factor as an effector of angiogenesis and tumor progression in hematological malignancies. *Leukemia*.

[69] Cocco E, Varughese J, Buza N (2011). Expression of Tissue factor in adenocarcinoma and squamous cell carcinoma of the uerine cervix: implications for immunotherapy with hI-con1, a factor VII-IgGFaac chimeric protein targeting tissue factor. *BMC Cancer*.

[70] Jude B, Zawadzki C, Susen S, Corseaux D (2005). Relevance of tissue factor in cardiovascular disease. *Archives des Maladies du Coeur et des Vaisseaux*.

[71] Mumford AD, McVey JH (2004). Tissue Factor in the myocardium: evidence of roles in haemostasis and inflammation. *Disease Markers*.

[72] Ray B, Chetter IC, Lee HLD, Ettelaie C, McCollum PT (2007). Plasma tissue factor is a predictor for restenosis after femoropopliteal angioplasty. *British Journal of Surgery*.

[73] So AK, Varisco PA, Kemkes-Matthes B (2003). Arthritis is linked to local and systemic activation of coagulation and fibrinolysis pathways. *Journal of Thrombosis and Haemostasis*.

[74] Santucci RA, Erlich J, Labriola J (2000). Measurement at tissue factor activity in whole blood. *Thrombosis and Haemostasis*.

[75] Berckmans RJ, Nieuwland R, Böing AN, Romijn FPHTM, Hack CE, Sturk A (2001). Cell-derived microparticles circulate in healthy humans and support low grade thrombin generation. *Thrombosis and Haemostasis*.

[76] Rand MD, Lock JB, Van't Veer C, Gaffney DP, Mann KG (1996). Blood clotting in minimally altered whole blood. *Blood*.

[77] Cawthern KM, Van’t Veer C, Lock JB, DiLorenzo ME, Branda RF, Mann KG (1998). Blood coagulation in hemophilia A and hemophilia C. *Blood*.

[78] Peyrou V, Lormeau JC, Hérault JP, Gaich C, Pfliegger AM, Herbert JM (1999). Contribution of erythrocytes to thrombin generation in whole blood. *Thrombosis and Haemostasis*.

[79] He R, Xiong S, He X (2001). The role of factor XI in a dilute thromboplastin assay of extrinsic coagulation pathway. *Thrombosis and Haemostasis*.

[80] Keularts IMLW, Zivelin A, Seligsohn U, Coenraad Hemker H, Béguin S (2001). The role of factor XI in thrombin generation induced by low concentrations of tissue factor. *Thrombosis and Haemostasis*.

[81] Gregory SA, Morrissey JH, Edgington TS (1989). Regulation of tissue factor gene expression in the monocyte procoagulant response to endotoxin. *Molecular and Cellular Biology*.

[82] Franco RF, De Jonge E, Dekkers PEP (2000). The in vivo kinetics of tissue factor messenger RNA expression during human endotoxemia: relationship with activation of coagulation. *Blood*.

[83] Egorina EM, Sovershaev MA, Bjørkøy G (2005). Intracellular and surface distribution of monocyte tissue factor: application to intersubject variability. *Arteriosclerosis, Thrombosis, and Vascular Biology*.

[84] Owens AP, Passam FH, Antoniak S, Marshall SM, McDaniel AL, Rudel L (2012). Monocyte tissue factor-dependent activation of coagulation in hypercholesterolemic mice and monkeys is inhibited by simvastatin. *The Journal of Clinical Investigation*.

[85] Meisel SR, Xu XP, Edgington TS (2011). Dose-dependent modulation of tissue factor protein and procoagulant activity in human monocyte-derived macrophages by oxidized low density lipoprotein. *Journal of Atherosclerosis and Thrombosis*.

[86] Esmon CT, Fukudome K, Mather T (1999). Inflammation, sepsis, and coagulation. *Haematologica*.

[87] Christersson C, Johnell M, Siegbahn A (2008). Tissue factor and IL8 production by P-selectin-dependent platelet-monocyte aggregates in whole blood involves phosphorylation of Lyn and is inhibited by IL10. *Journal of Thrombosis and Haemostasis*.

[88] Sovershaev MA, Egorina EM, Osterud B, Hansen JB (2012). Evidence for direct transfer of tissue factor from monocytes to platelets in whole blood. *Blood Coagulation & Fibrinolysis*.

[89] Hayashi M, Takeshita K, Inden Y (2011). Platelet activation and induction of tissue factor in acute and chronic atrial fibrillation: involvement of mononuclear cell-platelet interaction. *Thrombosis Research*.

[90] Zillmann A, Luther T, Müller I (2001). Platelet-associated tissue factor contributes to the collagen-triggered activation of blood coagulation. *Biochemical and Biophysical Research Communications*.

[91] Müller I, Klocke A, Alex M (2003). Intravascular tissue factor initiates coagulation via circulating microvesicles and platelets. *The FASEB Journal*.

[92] Panes O, Matus V, Sáez CG, Quiroga T, Pereira J, Mezzano D (2007). Human platelets synthesize and express functional tissue factor. *Blood*.

[93] Schwertz H, Tolley ND, Foulks JM (2006). Signal-dependent splicing of tissue factor pre-mRNA modulates the thrombogenecity of human platelets. *Journal of Experimental Medicine*.

[94] Camera M, Frigerio M, Toschi V (2003). Platelet activation induces cell-surface immunoreactive tissue factor expression, which is modulated differently by antiplatelet drugs. *Arteriosclerosis, Thrombosis, and Vascular Biology*.

[95] Camera M, Brambilla M, Facchinetti L (2012). Tissue factor and atherosclerosis: not only vessel wall-derived TF, but also platelet-associated TF. *Thrombosis Research*.

[96] Brambilla M, Camera M, Colnago D (2008). Tissue factor in patients with acute coronary syndromes: expression in platelets, leukocytes, and platelet-leukocyte aggregates. *Arteriosclerosis, Thrombosis, and Vascular Biology*.

[97] Bouchard BA, Mann KG, Butenas S (2010). No evidence for tissue factor on platelets. *Blood*.

[98] Osterud B, Bjorklid E (2006). Sources of tissue factor. *Seminars in Thrombosis and Hemostasis*.

[99] Osterud B, Bjorklid E (2012). Tissue factor in blood cells and endothelial cells. *Frontiers in Bioscience*.

[100] Camera M, Brambilla M, Toschi V, Tremoli E (2010). Tissue factor expression on platelets is a dynamic event. *Blood*.

[101] Bouchard BA, Krudysz-Amblo J, Butenas S (2012). Platelet tissue factor is not expressed transiently after platelet activation. *Blood*.

[102] Maugeri N, Brambilla M, Camera M (2006). Human polymorphonuclear leukocytes produce and express functional tissue factor upon stimulation. *Journal of Thrombosis and Haemostasis*.

[103] Ritis K, Doumas M, Mastellos D (2006). A novel C5a receptor-tissue factor cross-talk in neutrophils links innate immunity to coagulation pathways. *Journal of Immunology*.

[104] Moosbauer C, Morgenstern E, Cuvelier SL (2007). Eosinophils are a major intravascular location for tissue factor storage and exposure. *Blood*.

[105] Østerud B, Rao LVM, Olsen JO (2000). Induction of tissue factor expression in whole blood: lack of evidence for the presence of tissue factor expression in granulocytes. *Thrombosis and Haemostasis*.

[106] Sovershaev MA, Lind KF, Devold H (2008). No evidence for the presence of tissue factor in high-purity preparations of immunologically isolated eosinophils. *Journal of Thrombosis and Haemostasis*.

[107] Østerud B (2001). The role of platelets in decrypting monocyte tissue factor. *Seminars in Hematology*.

[108] Freyssinet JM, Toti F (2010). Formation of procoagulant microparticles and properties. *Thrombosis Research*.

[109] Théry C, Ostrowski M, Segura E (2009). Membrane vesicles as conveyors of immune responses. *Nature Reviews Immunology*.

[110] Aleman MM, Gardiner C, Harrison P, Wolberg AS (2011). Differential contributions of monocyte- and platelet-derived microparticles towards thrombin generation and fibrin formation and stability. *Journal of Thrombosis and Haemostasis*.

[111] Basavaraj MG, Braekkan SK, Brodin E, Osterud B, Hansen JB (2011). Monocyte count and procoagulant functions are associated with risk of venous thromboembolism: the Tromso study. *Journal of Thrombosis and Haemostasis*.

[112] Breimo ES, Østerud B (2005). Generation of tissue factor-rich microparticles in an ex vivo whole blood model. *Blood Coagulation and Fibrinolysis*.

[113] Mobarrez F, Antovic J, Egberg N (2010). A multicolor flow cytometric assay for measurement of platelet-derived microparticles. *Thrombosis Research*.

[114] Osterud B (2010). Tissue factor expression in blood cells. *Thrombosis Research*.

[115] Key NS (2010). Analysis of tissue factor positive microparticles. *Thrombosis Research*.

[116] Garcia Rodriguez P, Eikenboom HCJ, Tesselaar MET (2010). Plasma levels of microparticle-associated tissue factor activity in patients with clinically suspected pulmonary embolism. *Thrombosis Research*.

[117] Owen BAL, Xue A, Heit JA, Owen WG (2011). Procoagulant activity, but not number, of microparticles increases with age and in individuals after a single venous thromboembolism. *Thrombosis Research*.

[118] Langer F, Spath B, Haubold K (2008). Tissue factor procoagulant activity of plasma microparticles in patients with cancer-associated disseminated intravascular coagulation. *Annals of Hematology*.

[119] Manly DA, Wang J, Glover SL (2010). Increased microparticle tissue factor activity in cancer patients with Venous Thromboembolism. *Thrombosis Research*.

[120] Campello E, Spiezia L, Radu CM (2011). Endothelial, platelet, and tissue factor-bearing microparticles in cancer patients with and without venous thromboembolism. *Thrombosis Research*.

[121] Auwerda JJA, Yuana Y, Osanto S (2011). Microparticle-associated tissue factor activity and venous thrombosis in multiple myeloma. *Thrombosis and Haemostasis*.

[122] Haubold K, Rink M, Spath B (2009). Tissue factor procoagulant activity of plasma microparticles is increased in patients with early-stage prostate cancer. *Thrombosis and Haemostasis*.

[123] Gross PL, Vaezzadeh N (2010). Tissue factor microparticles and haemophilia. *Thrombosis Research*.

[124] Bogdanov VY, Balasubramanian V, Hathcock J, Vele O, Lieb M, Nemerson Y (2003). Alternatively spliced human tissue factor: a circulating, soluble, thrombogenic protein. *Nature Medicine*.

[125] Censarek P, Bobbe A, Grandoch M, Schrör K, Weber AA (2007). Alternatively spliced human tissue (asHTF) is not pro-coagulant. *Thrombosis and Haemostasis*.

[126] Hobbs JE, Zakarija A, Cundiff DL (2007). Alternatively spliced human tissue factor promotes tumor growth and angiogenesis in a pancreatic cancer tumor model. *Thrombosis Research*.

[127] Böing AN, Hau CM, Sturk A, Nieuwland R (2009). Human alternatively spliced tissue factor is not secreted and does not trigger coagulation. *Journal of Thrombosis and Haemostasis*.

[128] Szotowski B, Antoniak S, Poller W, Schultheiss HP, Rauch U (2005). Procoagulant soluble tissue factor is released from endothelial cells in response to inflammatory cytokines. *Circulation Research*.

[129] Boltzen U, Eisenreich A, Antoniak S (2012). Alternatively spliced tissue factor and full-length tissue factor protect cardiomyocytes against TNF-alpha-induced apoptosis. *Journal of Molecular and Cellular Cardiology*.

[130] van den Berg YW, van den Hengel LG, Myers HR (2009). Alternatively spliced tissue factor induces angiogenesis through integrin ligation. *Proceedings of the National Academy of Sciences of the United States of America*.

[131] Godby RC, Van Den Berg YW, Srinivasan R (2012). Nonproteolytic properties of murine alternatively spliced tissue factor: implications for integrin-mediated signaling in murine models. *Molecular Medicine*.

[132] van den Berg YW, Versteeg HH (2010). Alternatively spliced tissue factor: a crippled protein in coagulation or a key player in non-haemostatic processes?. *Hamostaseologie*.

[133] Khan MMH, Hattori T, Niewiarowski S, Edmunds LH, Colman RW (2006). Truncated and microparticle-free soluble tissue factor bound to peripheral monocytes preferentially activate factor VII. *Thrombosis and Haemostasis*.

[134] Østerud B, Breimo ES, Olsen JO (2008). Blood borne tissue factor revisited. *Thrombosis Research*.

[135] Parhami-Seren B, Butenas S, Krudysz-Amblo J, Mann KG (2006). Immunologic quantitation of tissue factors. *Journal of Thrombosis and Haemostasis*.

[136] Bogdanov VY, Cimmino G, Tardos JG, Tunstead JR, Badimon JJ (2009). Assessment of plasma tissue factor activity in patients presenting with coronary artery disease: limitations of a commercial assay. *Journal of Thrombosis and Haemostasis*.

[137] Bis J, Vojacek J, Dusek J (2009). Time-course of tissue factor plasma level in patients with acute coronary syndrome. *Physiological Research*.

[138] Butenas S, Undas A, Gissel MT, Szuldrzynski K, Zmudka K, Mann KG (2008). Factor Xla and tissue factor activity in patients with coronary artery disease. *Thrombosis and Haemostasis*.

[139] Paborsky LR, Tate KM, Harris RJ (1989). Purification of recombinant human tissue factor. *Biochemistry*.

[140] Waxman E, Ross JBA, Laue TM (1992). Tissue factor and its extracellular soluble domain: the relationship between intermolecular association with factor VIIa and enzymatic activity of the complex. *Biochemistry*.

[141] Paborsky LR, Harris RJ (1990). Post-translational modifications of recombinant human tissue factor. *Thrombosis Research*.

[142] Chargaff E, Bendich A, Cohen S (1944). The thromboplastic protein: structure, properties, disintegration. *Journal of Biological Chemistry*.

[143] Bjorklid E, Storm E (1977). Purification and some properties of the protein component of tissue thromboplastin from human brain. *Biochemical Journal*.

[144] Rickles FR, Contrino J, Kreutzer DL (1996). Tissue factor expression in normal and abnormal mammary gland—reply. *Nature Medicine*.

[145] Stone MJ, Ruf W, Miles DJ, Edgington TS, Wright PE (1995). Recombinant soluble human tissue factor secreted by *Saccharomyces cerevisiae* and refolded from *Escherichia coli* inclusion bodies: glycosylation of mutants, activity and physical characterization. *Biochemical Journal*.

[146] Pitlick FA (1975). Concanavalin A inhibits tissue factor coagulant activity. *Journal of Clinical Investigation*.

[147] Shands JW (1985). Macrophage factor X activator formation: metabolic requirements for synthesis of components. *Blood*.

[148] Bona R, Lee E, Rickles F (1987). Tissue factor apoprotein: intracellular transport and expression in shed membrane vesicles. *Thrombosis Research*.

[149] Cohen P (1982). The role of protein phosphorylation in neural and hormonal control of cellular activity. *Nature*.

[150] Zioncheck TF, Roy S, Vehar GA (1992). The cytoplasmic domain of tissue factor is phosphorylated by a protein kinase C-dependent mechanism. *Journal of Biological Chemistry*.

[151] Mody RS, Carson SD (1997). Tissue factor cytoplasmic domain peptide is multiply phosphorylated in vitro. *Biochemistry*.

[152] Dorfleutner A, Ruf W (2003). Regulation of tissue factor cytoplasmic domain phosphorylation by palmitoylation. *Blood*.

[153] Car BD, Slauson DO, Dore M, Suyemoto MM (1990). Endotoxin-mediated bovine alveolar macrophage procoagulant induction is dependent on protein kinase C activation. *Inflammation*.

[154] Nishizuka Y (1986). Studies and perspectives of protein kinase C. *Science*.

[155] Versteeg HH, Hoedemaeker I, Diks SH (2000). Factor VIIa/tissue factor-induced signaling via activation of src-like kinases, phosphatidylinositol 3-kinase, and Rac. *Journal of Biological Chemistry*.

[156] Poulsen LK, Jacobsen N, Sørensen BB (1998). Signal transduction via the mitogen-activated protein kinase pathway induced by binding of coagulation factor VIIa to tissue factor. *Journal of Biological Chemistry*.

[157] Rydén L, Grabau D, Schaffner F, Jönsson PE, Ruf W, Belting M (2010). Evidence for tissue factor phosphorylation and its correlation with protease-activated receptor expression and the prognosis of primary breast cancer. *International Journal of Cancer*.

[158] Magee AI, Courtneidge SA (1985). Two classes of fatty acid acylated proteins exist in eukaryotic cells. *The EMBO Journal*.

[159] Schroeder R, London E, Brown D (1994). Interactions between saturated acyl chains confer detergent resistance on lipids and glycosylphosphatidylinositol (GPI)-anchored proteins: GPI-anchored proteins in liposomes and cells show similar behavior. *Proceedings of the National Academy of Sciences of the United States of America*.

[160] Bach R, Konigsberg WH, Nemerson Y (1988). Human tissue factor contains thioester-linked palmitate and stearate on the cytoplasmic half-cystine. *Biochemistry*.

[161] Broze GJ, Leykam JE, Schwartz BD, Miletich JP (1985). Purification of human brain tissue factor. *Journal of Biological Chemistry*.

[162] Guha A, Bach R, Konigsberg W, Nemerson Y (1986). Affinity purification of human tissue factor: interaction of factor VII and tissue factor in detergent micelles. *Proceedings of the National Academy of Sciences of the United States of America*.

[163] Carson SD, Ross SE, Gramzinski RA (1988). Protein co-isolated with human tissue factor impairs recovery of activity. *Blood*.

[164] Edidin M (2003). The state of lipid rafts: from model membranes to cells. *Annual Review of Biophysics and Biomolecular Structure*.

[165] Fortin JP, Rivard GE, Adam A, Marceau F (2005). Studies on rabbit natural and recombinant tissue factors: intracellular retention and regulation of surface expression in cultured cells. *American Journal of Physiology*.

[166] Mandal SK, Pendurthi UR, Rao LVM (2006). Cellular localization and trafficking of tissue factor. *Blood*.

[167] Sevinsky JR, Mohan Rao LV, Ruf W (1996). Ligand-induced protease receptor translocation into caveolae: a mechanism for regulating cell surface proteolysis of the tissue factor-dependent coagulation pathway. *Journal of Cell Biology*.

[168] Mandal SK, Iakhiaev A, Pendurthi UR, Rao LVM (2005). Acute cholesterol depletion impairs functional expression of tissue factor in fibroblasts: modulation of tissue factor activity by membrane cholesterol. *Blood*.

[169] Dietzen DJ, Page KL, Tetzloff TA (2004). Lipid rafts are necessary for tonic inhibition of cellular tissue factor procoagulant activity. *Blood*.

[170] Del Conde I, Shrimpton CN, Thiagarajan P, López JA (2005). Tissue-factor-bearing microvesicles arise from lipid rafts and fuse with activated platelets to initiate coagulation. *Blood*.

[171] Rao LVM, Pendurthi UR (2012). Regulation of tissue factor coagulant activity on cell surfaces. *Journal of Thrombosis and Haemostasis*.

[172] Bach RR (2006). Tissue factor encryption. *Arteriosclerosis, Thrombosis, and Vascular Biology*.

[173] Ruf W, Rehemtulla A, Miles DJ, Edgington TS (1991). The integrity of the cysteine 186-cysteine 209 bond of the second disulfide loop of tissue factor is required for binding of factor VII. *Journal of Biological Chemistry*.

[174] Chen VM, Ahamed J, Versteeg HH, Berndt MC, Ruf W, Hogg PJ (2006). Evidence for activation of tissue factor by an allosteric disulfide bond. *Biochemistry*.

[175] Kaneko H, Kakkar VV, Scully MF (1994). Mercury compounds induce a rapid increase in procoagulant activity of monocyte-like U937 cells. *British Journal of Haematology*.

[176] Ahamed J, Versteeg HH, Kerver M (2006). Disulfide isomerization switches tissue factor from coagulation to cell signaling. *Proceedings of the National Academy of Sciences of the United States of America*.

[177] Raturi A, Ruf W (2010). Effect of protein disulfide isomerase chaperone activity inhibition on tissue factor activity. *Journal of Thrombosis and Haemostasis*.

[178] Versteeg HH, Ruf W (2007). Tissue factor coagulant function is enhanced by protein-disulfide isomerase independent of oxidoreductase activity. *Journal of Biological Chemistry*.

[179] Reinhardt C, Von Brühl ML, Manukyan D (2008). Protein disulfide isomerase acts as an injury response signal that enhances fibrin generation via tissue factor activation. *Journal of Clinical Investigation*.

[180] Manukyan D, von Bruehl ML, Massberg S, Engelmann B (2008). Protein disulfide isomerase as a trigger for tissue factor-dependent fibrin generation. *Thrombosis Research*.

[181] Kothari H, Sen P, Pendurthi UR, Rao LVM (2008). Bovine protein disulfide isomerase-enhanced tissue factor coagulant function: is phospholipid contaminant in it the real culprit?. *Blood*.

[182] Persson E (2008). Protein disulfide isomerase has no stimulatory chaperone effect on factor X activation by factor VIIa-soluble tissue factor. *Thrombosis Research*.

[183] Pendurthi UR, Rao LVM (2008). Response: tissue factor de-encryption: the cell model system. *Blood*.

[184] Popescu NI, Lupu C, Lupu F (2010). Extracellular protein disulfide isomerase regulates coagulation on endothelial cells through modulation of phosphatidylserine exposure. *Blood*.

[185] Popescu NI, Lupu C, Lupu F (2010). Role of PDI in regulating tissue factor: FVIIa activity. *Thrombosis Research*.

[186] Pendurthi UR, Ghosh S, Mandal SK, Rao LVM (2007). Tissue factor activation: is disulfide bond switching a regulatory mechanism?. *Blood*.

[187] Bach RR, Monroe D (2009). What is wrong with the allosteric disulfide bond hypothesis?. *Arteriosclerosis, Thrombosis, and Vascular Biology*.

[188] Kothari H, Nayak RC, Rao LVM, Pendurthi UR (2010). Cystine 186-cystine 209 disulfide bond is not essential for the procoagulant activity of tissue factor or for its de-encryption. *Blood*.

[189] van den Hengel LG, Kocaturk B, Reitsma PH, Ruf W, Versteeg HH (2011). Complete abolishment of coagulant activity in monomeric disulfide-deficient tissue factor. *Blood*.

[190] Ruf W, Versteeg HH (2010). Tissue factor mutated at the allosteric Cys186-Cys209 disulfide bond is severely impaired in decrypted procoagulant activity. *Blood*.

[191] Schmidt B, Ho L, Hogg PJ (2006). Allosteric disulfide bonds. *Biochemistry*.

[192] Bednar RA, Fried WB, Lock YW, Pramanik B (1989). Chemical modification of chalcone isomerase by mercurials and tetrathionate. Evidence for a single cysteine residue in the active site. *Journal of Biological Chemistry*.

[193] Weber GJ, Mehr AP, Sirota JC (2006). Mercury and zinc differentially inhibit shark and human CFTR orthologues: involvement of shark cysteine 102. *American Journal of Physiology*.

[194] Hatahet F, Ruddock LW (2007). Substrate recognition by the protein disulfide isomerases. *FEBS Journal*.

[195] Hogg PJ (2003). Disulfide bonds as switches for protein function. *Trends in Biochemical Sciences*.

[196] Chen VM, Hogg PJ (2006). Allosteric disulfide bonds in thrombosis and thrombolysis. *Journal of Thrombosis and Haemostasis*.

[197] Jurk K, Lahav J, Vana H, Brodde MF, Nofer JR, Kehrel BE (2011). Extracellular protein disulfide isomerase regulates feedback activation of platelet thrombin generation via modulation of coagulation factor binding. *Journal of Thrombosis and Haemostasis*.

[198] Essex DW, Miller A, Swiatkowska M, Feinman RD (1999). Protein disulfide isomerase catalyzes the formation of disulfide-linked complexes of vitronectin with thrombin-antithrombin. *Biochemistry*.

[199] Van den Hengel LG, Osanto S, Reitsma PH, Versteeg H Murine tissue factor coagulant activity is critically dependent on the presence of an intact allosteric disulfide.

